# A Panel of 4 microRNAs Facilitates the Prediction of Left Ventricular Contractility after Acute Myocardial Infarction

**DOI:** 10.1371/journal.pone.0070644

**Published:** 2013-08-13

**Authors:** Yvan Devaux, Melanie Vausort, Gerry P. McCann, Dominic Kelly, Olivier Collignon, Leong L. Ng, Daniel R. Wagner, Iain B. Squire

**Affiliations:** 1 Laboratory of Cardiovascular Research, Centre de Recherche Public de la Santé, Luxembourg, Luxembourg; 2 Department of Cardiovascular Sciences, University of Leicester, and the National Institute for Health Research, Leicester Cardiovascular Biomedical Research Unit, Glenfield Hospital, Leicester, United Kingdom; 3 Competences Center for Methodology and Statistics, Centre de Recherche Public de la Santé, Luxembourg, Luxembourg; 4 Division of Cardiology, Centre Hospitalier, Luxembourg, Luxembourg; I2MC INSERM UMR U1048, France

## Abstract

**Background:**

Prediction of clinical outcome after acute myocardial infarction (AMI) is challenging and would benefit from new biomarkers. We investigated the prognostic value of 4 circulating microRNAs (miRNAs) after AMI.

**Methods:**

We enrolled 150 patients after AMI. Blood samples were obtained at discharge for determination of N-terminal pro-brain natriuretic peptide (Nt-proBNP) and levels of miR-16, miR-27a, miR-101 and miR-150. Patients were assessed by echocardiography at 6 months follow-up and the wall motion index score (WMIS) was used as an indicator of left ventricular (LV) contractility. We assessed the added predictive value of miRNAs against a multi-parameter clinical model including Nt-proBNP.

**Results:**

Patients with anterior AMI and elevated Nt-proBNP levels at discharge from the hospital were at high risk of subsequent impaired LV contractility (follow-up WMIS>1.2, n = 71). A combination of the 4 miRNAs (miR-16/27a/101/150) improved the prediction of LV contractility based on clinical variables (P = 0.005). Patients with low levels of miR-150 (odds ratio [95% confidence interval] 0.08 [0.01–0.48]) or miR-101 (0.19 [0.04–0.97]) and elevated levels of miR-16 (15.9 [2.63–95.91]) or miR-27a (4.18 [1.36–12.83]) were at high risk of impaired LV contractility. The 4 miRNA panel reclassified a significant proportion of patients with a net reclassification improvement of 66% (P = 0.00005) and an integrated discrimination improvement of 0.08 (P = 0.001).

**Conclusion:**

Our results indicate that panels of miRNAs may aid in prognostication of outcome after AMI.

## Introduction

Left ventricular (LV) remodelling develops in a significant proportion of patients after acute myocardial infarction (AMI) and is associated with a high mortality and morbidity [1]. Early identification of patients at risk of LV remodelling may facilitate prompt initiation and optimisation of evidence-based interventions and pharmacological therapies. A number of biomarkers are utilised in this context; the current gold-standard used to predict outcome after AMI, N-terminal pro-brain natriuretic peptide (Nt-proBNP), has important limitations in clinical practice, as concentrations fluctuate after AMI [2]. However, in patients with AMI, Nt-proBNP correlates with wall motion index score (WMIS), a measure of LV remodelling and dysfunction [2].

Since the discovery of their stability in the bloodstream [3], [4], microRNAs (miRNAs), short oligonucleotides which down-regulate gene expression, have been the focus of numerous biomarker studies. While the potential utility of miRNAs in the diagnosis of AMI has been addressed in several reports [5] including ours [6], [7], their prognostic value in this setting has received less attention. Interestingly, the temporal profile of circulating miRNAs is related to the development of LV remodelling after AMI [8], which suggested their potential utility as prognostic biomarkers.

A study by Widera et al. reported that plasma levels of cardiac-enriched miR-133a and miR-208b were associated with mortality in patients with acute coronary syndrome [9]. Nevertheless, this association lost its significance upon further adjustment with high-sensitivity troponin T. We observed an inverse correlation between initial levels of miR-208b and miR-499 and left ventricular ejection fraction at 4-months follow-up in patients with AMI [7]. However, neither miRNA was of independent prognostic value.

Using a systems-based approach and interaction network analysis, we previously identified 10 miRNAs likely to regulate the expression of genes associated with LV remodelling [10]. Based on the results of preliminary pilot studies, we sought to determine the prognostic value of a group of 4 miRNAs, miR-16/27a/101/150, in a prospective cohort of AMI patients.

## Materials and Methods

### Patients

We enrolled 150 patients with AMI (Table 1). The diagnosis of AMI was based on presentation with appropriate symptoms of myocardial ischemia, dynamic ST segment elevation, and increase in markers of myocyte necrosis (creatine kinase (CK) and troponin I (TnI)) to above twice the upper limit of the normal range. Venous blood samples for assay of miRNAs and Nt-proBNP were collected in EDTA-aprotinin tubes, immediately prior to discharge (day 3–4 after AMI). Samples were centrifuged within 30 minutes and plasma stored in aliquots at −80°C.

**Table 1:** Demographic and clinical features of AMI patients.

	All	Follow-up WMIS≤1.2	Follow-up WMIS>1.2	*P* 1
	(N = 150)	(N = 79)	(N = 71)	
Age, y (median-range)	64 (24–87)	61 (37–86)	65 (24–87)	0.56
Male, n (%)	116 (77%)	63 (80%)	53 (75%)	0.89
**Cardiovascular history/risk factors, n (%)**				
Smoker	60 (40%)	33 (42%)	27 (38%)	0.88
FH	59 (39%)	31 (42%)	28 (35%)	0.89
Angina	14 (28%)	5 (6%)	9 (13%)	0.35
Diabetes	24 (16%)	12 (15%)	12 (17%)	1
Hypertension	52 (35%)	26 (33%)	26 (37%)	1
Hypercholesterolaemia	40 (27%)	18 (23%)	22 (31%)	0.49
MI	12 (8%)	3 (4%)	9 (13%)	0.12
PCI	3 (2%)	3 (4%)	0 (0%)	0.30
CABG	1 (1%)	0 (0%)	1 (1%)	0.96
**Presentation, n (%)**				
STEMI	127 (85%)	62 (78%)	65 (92%)	0.60
Anterior infarct	59 (39%)	24 (30%)	35 (49%)	0.16
Thrombolysis	75 (50%)	42 (53%)	33 (46%)	0.74
**Serum markers during admission (median-range)**				
Troponin I (ng/mL)	9.83 (0.08–150)	5.90 (0.08–150)	19.95 (0.09–150)	0.001
CK (units/L)	985 (56–7384)	625 (56–3925)	1614 (123–7384)	<0.001
Nt-proBNP (ng/L)	2.80 (0.26–3.98)	2.53 (0.26–3.55)	3.16 (0.94–3.98)	<0.001
**Medications at admission, n (%)**				
Aspirin	21 (14%)	9 (11%)	12 (17%)	0.54
Clopidogrel	4 (3%)	3 (4%)	1 (1%)	0.71
Beta-blockers	24 (16%)	13 (16%)	11 (15%)	0.93
Calcium antagonists	22 (15%)	7 (9%)	15 (21%)	0.11
ACE inhibitors	17 (11%)	6 (8%)	11 (15%)	0.27
Angiotensin receptor blocker	9 (6%)	6 (8%)	3 (4%)	0.64
Statins	28 (19%)	13 (16%)	15 (21%)	0.69
**Medications at discharge, n (%)**				
Aspirin	134 (89%)	73 (92%)	61 (86%)	0.85
Clopidogrel	36 (24%)	23 (29%)	13 (18%)	0.30
Beta-blocker	142 95%)	75 (95%)	67 (94%)	0.93
ACE inhibitor	134 (89%)	71 (90%)	63 (89%)	0.95
Angiotensin receptor blocker	11 (7%)	5 (6%)	6 (8%)	0.88
Diuretic	15 (10%)	2 (3%)	13 (18%)	0.008
Statin	148 (99%)	78 (99%)	70 (99%)	0.91
**Endpoints at 6-months**				
Reinfarction, n (%)	15 (10%)	5 (6%)	10 (14%)	0.25
CHF, n (%)	11 (7%)	1 (1%)	10 (14%)	0.01
Death, n (%)	4 (3%)	1 (1%)	3 (4%)	0.56

1 For comparison between WMIS≤1.2 and WMIS>1.2.

ACE: angiotensin-converting enzyme; BNP: brain natriuretic peptide; CABG: coronary artery bypass grafting; CHF: congestive heart failure; CK: creatine kinase; FH: familial hypercholesterolemia; MI: myocardial infarction; PCI: percutaneous coronary intervention; STEMI: ST-elevation myocardial infarction.

The protocol was approved by the Derbyshire Research Ethics Committee and written informed consent was obtained from all subjects. The conduct of the study was in accordance with the Declaration of Helsinki.

Patients were admitted to Glenfield Hospital, Leicester, between September 2004 and March 2005, and were enrolled in a prospective study of LV remodelling after AMI [11]. Half of these patients were treated by thrombolysis and few received primary percutaneous coronary intervention (PPCI), which was not in routine use at this centre at this time. Clinically, no patient had unsuccessful reperfusion and none required transfer for rescue PPCI. Cardiac function was assessed by echocardiography, as described [11], conducted by a single operator (DK) at discharge and at a median of 176 days (range 138–262 days) after AMI. Left ventricular contractility was evaluated by the LV wall motion index score (WMIS), using a standard 16-segment model from parasternal long- and short-axis and apical two- and four-chamber views. Each LV segment was scored as 0, hyperkinetic; 1, normal; 2, hypokinetic; 3, akinetic; 4, dyskinetic. The total was divided by the number of segments analysed to give an overall score, with higher values indicating more impaired LV contractility. Using this methodology, WMIS = 1.2 is equivalent to LV ejection fraction of 40%, and this value was used to dichotomize patients into those with impaired (WMIS>1.2), and preserved (WMIS≤1.2) LV contractility at follow-up.

### Plasma miRNAs determination

Total RNA was extracted from plasma using miRVana PARIS isolation kit (Applied Biosystems, Lennik, Belgium) without enrichment for small RNAs. A mix of 3 spiked-in synthetic C elegans miRNAs were added to plasma samples to correct for extraction efficiency. Potential contaminating genomic DNA was removed by DNase (Qiagen, Venlo, The Netherlands). Reverse transcription of RNA was achieved with the miScript reverse transcription kit (Qiagen). The resulting cDNA was diluted 10-fold before amplification by quantitative PCR with SYBR-green PCR kit and miRNA-specific miScript primer set (Qiagen). Expression levels were calculated with the formula [2 exp (mean Ct spiked-in controls - Ct target miRNA)].

### Nt-proBNP assay

Plasma levels of Nt-proBNP at discharge were determined using a non-competitive assay, as described [12]. Detection limit of the assay was 14.4 fmol/mL. Intra- and inter- coefficients of variation were 2.3% 4.8%, respectively. No cross-reactivity with ANP, BNP, or CNP was detected.

### Statistical analysis

#### Patient characteristics

Comparisons of demographic features and echo parameters between groups of patients with (WMIS>1.2) and without (WMIS≤1.2) impaired LV contractility were performed by Chi-square test for categorical data. For continuous data, comparisons between two groups were performed with t-test for Gaussian data and the Mann-Whitney test on ranks for non-normally distributed data. Normality was assessed with the Shapiro-Wilk test. Analyses were carried out using SigmaPlot v 11.0. For all comparisons, a p<0.05 was considered statistically significant.

#### Prediction analyses

Prediction analyses were performed with R version 2.13.1 with Hmisc, aod, lmtest and AER packages. A p-value was considered significant when lower than 0.05. Clinical features were coded as 1 for presence and 0 for absence. Male was chosen as the reference level for sex in regression models. No data were missing thus no imputation was required.

#### Model fitting

WMIS was first dichotomized into two groups (WMIS≤1.2 and WMIS>1.2), which were analysed by logistic regression (models 1 and 2). A patient was classified as WMIS>1.2 when its probability was ≥0.5 and as WMIS≤1.2 otherwise. WMIS was then treated as a continuous variable (models 3 and 4). Since more than a third of the patients had a WMIS value of 1 (the remaining patients having greater values), a left censored tobit regression [13] was performed to model WMIS with different sets of predictors.

Model parameter estimates were tested for nullity using a Wald Chi-square test in logistic regression and a Z test in censored regression. Residuals were analysed graphically both to detect nonlinear relationships between each variable in a model and WMIS, and to check normality assumptions for tobit regression. For logistic regression, odd ratios (OR) and 95% confidence intervals (CI) were obtained by exponential transformation of the slope statistics.

#### Best model selection

To determine which miRNA or combination of miRNAs had the maximal added value, all 15 possible combinations of miRNAs among the 4 miRNAs measured were generated and successively added to the reference model containing clinical parameters and Nt-proBNP. For each model, a Wald Chi-square test was used to assess the global effect of explanatory variables on WMIS. The added value of miRNAs was tested for significance using the likelihood ratio test (LRT). In the dichotomous case, the continuous net reclassification improvement (NRI) and integrated discrimination improvement (IDI) [14] were evaluated and tested for nullity. The final model was finally selected by minimizing the Akaike Information Criterion (AIC) which is penalized by the number of variables added in the model to avoid over-fitting.

#### Model validation

Bootstrap internal validation [15] was used to correct all measures of model performance for over-fitting. For each bootstrap sample (i.e. a random sample of individuals with the same size as the original sample where a given patient can appear several times), the whole model selection process was performed again to select the best model according to the AIC criterion; the original sample was then tested with this model. In order to evaluate over-fitting, NRI and IDI were computed with the test (i.e. original) set and subtracted to the same measures computed with the bootstrap sample to evaluate optimism. Afterwards over-fitting was averaged across 150 bootstrap replications and finally subtracted to the measures obtained with the original sample as a training set.

#### Borderline patients classification

Borderline patients were defined as having 1<WMIS<1.4. To determine whether miRNAs improved the classification of these patients, cross-validation was performed by successively omitting these patients one by one during logistic regression. Sensitivity, specificity, positive and negative predictive values were then computed and compared exclusively for those patients between both models.

## Results

### Patient characteristics

Table 1 shows the demographic features of the patients. The vast majority presented with ST-elevation AMI (STEMI). Among the 150 patients enrolled, LV contractility at follow-up was impaired (WMIS>1.2) in 71 (47%) and preserved (WMIS≤1.2) in 79 (53%). Compared to patients with preserved LV contractility, those with impaired contractility had higher levels of troponin I, creatine kinase and Nt-proBNP at discharge. Diuretics were more often prescribed during the index admission in these patients, who also had higher risk of developing congestive heart failure during follow-up (Table 1).

Table 2 shows echocardiographic parameters of LV function, at discharge and at 6-months follow-up. Patients with impaired LV contractility at follow-up had lower EF and higher LV volumes and diameters, both at discharge from the hospital and at 6 months, compared to patients with preserved LV contractility.

**Table 2:** Echo parameters of AMI patients.

	All	Follow-up WMIS≤1.2	Follow-up WMIS>1.2	*P* 1
	(N = 150)	(N = 79)	(N = 71)	
**Pre-discharge echo (median-range)**				
LVEF (%)	44 (15–75)	50 (22–75)	37 (15–61)	<0.001
LVEDV (mL)	89 (36–201)	83 (36–159)	94 (36–201)	0.005
LVESV (mL)	46 (20–132)	39 (21–86)	56 (20–132)	<0.001
LVIDd (cm)	4.8 (2.9–6.6)	4.6 (2.9–6)	5.2 (3.7–8.1)	0.006
LVIDs (cm)	3.6 (1.8–5.5)	3.35 (1.8–5.2)	4.1 (1.7–7)	<0.001
WMIS	1.31 (1–2.38)	1.06 (1–1.2)	1.74 (1.25–2.38)	<0.001
**Follow-up echo (median-range)**				
LVEF (%)	48 (16–74)	52 (37–74)	40 (16–69)	<0.001
LVEDV (mL)	87 (40–208)	80 (45–163)	96 (40–208)	<0.001
LVESV (mL)	45 (17–141)	38 (17–89)	58 (17–141)	<0.001
LVIDd (cm)	4.9 (3–8.1)	4.8 (3–6.3)	5.2 (3.7–8.1)	<0.001
LVIDs (cm)	3.6 (1.6–7)	3.4 (1.6–4.9)	4.1 (1.7–7)	<0.001
WMIS	1.19 (1–2.38)	1 (1–1.2)	1.5 (1.25–2.38)	<0.001

1 For comparison between WMIS≤1.2 and WMIS>1.2.

LVEDV: left ventricular end-diastolic volume; LVESV: left ventricular end-systolic volume; LVEF: left ventricular ejection fraction; LVIDd: left ventricular internal diameter, diastole; LVIDs: left ventricular internal diameter, systole.

### Prediction of LV contractility

Logistic regression analyses were performed to investigate the association between miRNAs measured prior to discharge and the later development of impaired LV contractility. Two multivariable models were built. The first model ( = model 1) included the following parameters: age, gender, smoking habit, diabetes, hypertension, hypercholesterolemia, previous MI, infarct type (STEMI vs NSTEMI), infarct territory (anterior vs inferior), and Nt-proBNP level at discharge. The second model ( = model 2) included all the parameters of model 1 with the addition of expression values the 4 miRNAs.

**Figure 1:**
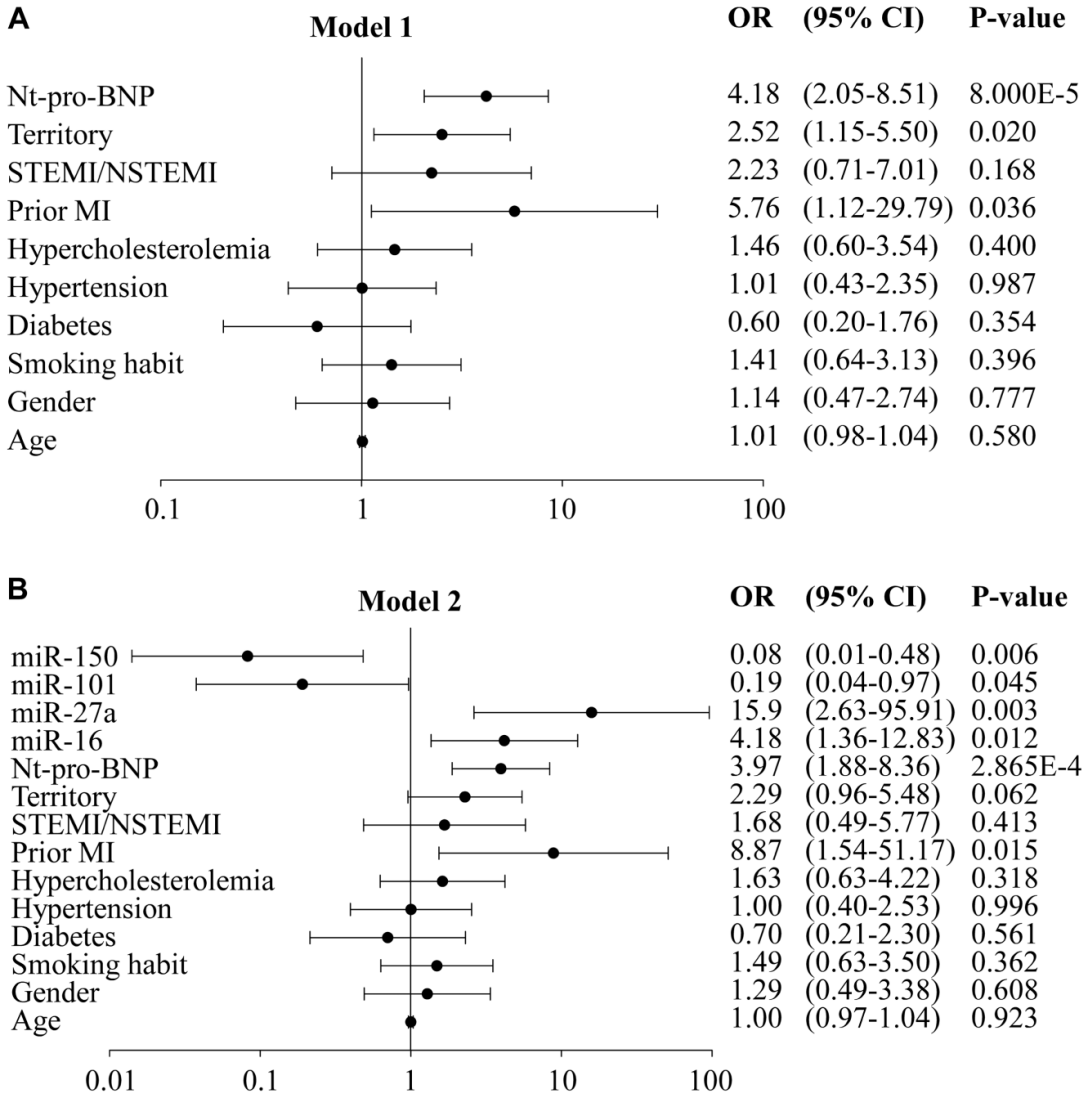
Odds ratios for clinical parameters, Nt-proBNP and miRNAs. Nt-proBNP and miRNAs were measured at discharge from the hospital and LV contractility was evaluated by WMIS at 6-months follow-up. Patients were dichotomized according to WMIS using a threshold value of 1.2. Patients with WMIS≤1.2 had preserved LV contractility (n = 79) and patients with WMIS>1.2 had impaired LV contractility (n = 71). Logistic regression models were built to determine the increased risk of impaired LV contractility. A. Model 1 is a multivariable model including indicated clinical parameters and Nt-proBNP. B. Model 2 is a multivariable model including the variables of model 1 and the expression values of miR-16/27a/101/150. CI: confidence interval; OR: odd ratio. Note: X axis is in log scale.

Odds ratios for both models are shown in Figure 1. Patients with anterior STEMI, history of AMI and elevated Nt-proBNP were at high risk of impaired LV contractility (Figure 1A). Plasma levels of each of the 4 miRNAs were associated with the presence or absence of LV dysfunction. Patients with low levels of miR-150/101 or elevated levels of miR-16/27a were at increased risk of impaired LV contractility (Figure 1B).

We next determined the added value of combinations of miRNAs. The AIC was used in this analysis since this criterion is adjusted by the number of variables, in contrast to AUC, the use of which involves the possibility of better prediction due to greater number of variables included in the model. Lower AIC is indicative of better model fit. As shown in Table 3, adding the 4 miRNAs to the model with clinical parameters and Nt-proBNP (model 1) resulted in a statistically significant reduction in the AIC from 188.269 to 181.432 (P = 0.005). miR-27a/150 was the smallest combination of miRNAs which generated added value (P = 0.046).

**Table 3:** Added value of combinations of miRNAs (logistic regression).

miRNA added to model 1	Wald chi square test P-value	AIC	LRT P-value
None	0.003	188.269	
miR-16	0.003	188.381	0.169
miR-27a	0.003	186.591	0.055
miR-101	0.004	189.476	0.373
miR-150	0.004	190.261	0.931
miR-16+miR-27a	0.005	188.245	0.134
miR-16+miR-101	0.006	190.332	0.380
miR-16+miR-150	0.003	187.753	0.105
miR-27a+miR-101	0.005	186.842	0.066
miR-27a+miR-150	0.004	186.117	0.046
miR-101+miR-150	0.006	191.080	0.552
miR-16+miR-27a+miR-101	0.007	187.837	0.092
miR-16+miR-27a+miR-150	0.003	183.838	0.015
miR-16+miR-101+miR-150	0.005	189.380	0.180
miR-27a+miR-101+miR-150	0.006	186.389	0.049
miR-16+miR-27a+miR-101+miR-150	0.003	181.432	0.005

Shown are the results of all combinations of miRNAs added to model 1. The Wald chi square test indicates the overall significance of the model. The likelihood ratio test (LRT) compares the fit of a model with miRNAs to model 1. AIC: Akaike information criteria.

**Figure 2:**
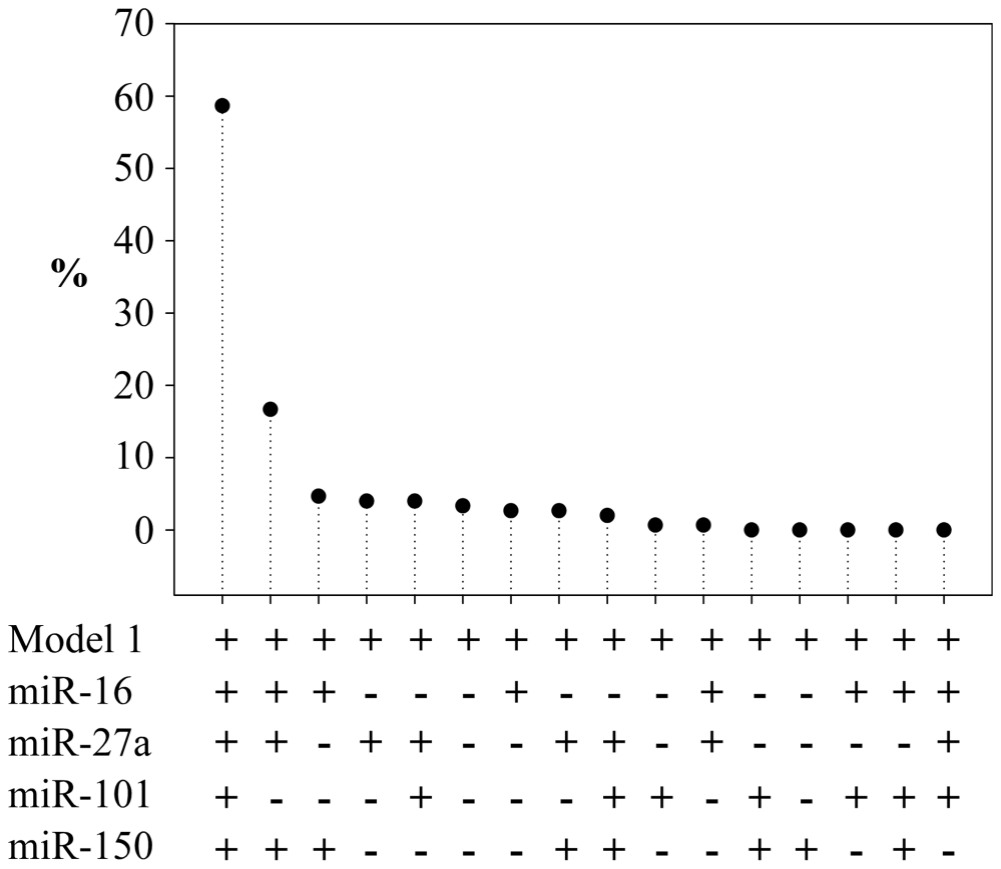
Bootstrap internal validation (logistic regression). Represented is the percentage of times a combination of miRNAs was selected as providing the best improvement of the prediction of model 1 over 150 bootstrap iterations.

Bootstrap internal validation was used to evaluate the robustness of the models with miRNAs (Figure 2). The principle of this method is to calculate the predictive value of the model after re-sampling patients from the original sample. This approach was also used to evaluate the robustness of miRNAs selection process and confirmed that the 4 miRNA panel provided the optimal improvement of prediction (59% of bootstrap samples). Inclusion in the model of reperfusion therapy did not alter meaningfully the relationship between miRNA expression and LV function (data not shown).

### Reclassification analyses

The continuous version of the NRI and the IDI were computed to determine the ability of miRNAs to correctly reclassify patients misclassified by model 1 (Table 4). These are indexes of the change in classification of patients from one category of WMIS to another category (≤1.2 or >1.2). The 4 miRNA panel was able to reclassify a significant proportion of patients, as attested by a NRI of 66% (P = 0.00005) and an IDI of 0.08 (P = 0.001). After bootstrap validation, NRI and IDI were 50% and 0.05, respectively. Several combinations of miRNAs also provided statistically significant reclassifications, such as miR-16/150, miR-27a/150, miR-16/27a/150, or miR-27a/101/150. However, no single miRNA had a statistically significant reclassification capability.

**Table 4:** Reclassification analyses (logistic regression).

miRNA added to model 1	NRI	95% CI	NRI P-value	IDI	95% CI	IDI P-value
miR-16	0.179	−0.142–0.499	0.275	0.010	−0.007–0.027	0.243
miR-27a	0.263	−0.057–0.584	0.108	0.017	−0.007–0.040	0.162
miR-101	0.181	−0.139–0.502	0.267	0.004	−0.007–0.015	0.453
miR-150	0.120	−0.201–0.440	0.464	1.53E-04	−0.001–0.001	0.774
miR-16+miR-27a	0.314	−0.007–0.634	0.055	0.019	−0.005–0.044	0.125
miR-16+miR-101	0.125	−0.195–0.446	0.444	0.010	−0.007–0.027	0.232
miR-16+miR-150	0.331	0.010–0.651	0.043	0.028	0.002–0.053	0.033
miR-27a+miR-101	0.379	0.058–0.699	0.021	0.024	−0.004–0.053	0.087
miR-27a+miR-150	0.646	0.326–0.967	0.00008	0.031	0.001–0.061	0.046
miR-101+miR-150	0.213	−0.108–0.533	0.194	0.007	−0.006–0.021	0.296
miR-16+miR-27a+miR-101	0.474	0.154–0.795	0.004	0.030	−0.001–0.060	0.054
miR-16+miR-27a+miR-150	0.415	0.095–0.736	0.011	0.056	0.018–0.095	0.004
miR-16+miR-101+miR-150	0.257	−0.063–0.578	0.115	0.030	0.004–0.056	0.025
miR-27a+miR-101+miR-150	0.514	0.193–0.834	0.002	0.039	0.005–0.072	0.023
miR-16+miR-27a+miR-101+miR-150	0.663	0.342–0.983	0.00005	0.077	0.032–0.122	0.001

Shown are the results of all combinations of miRNAs added to model 1. The continuous version of the net reclassification improvement (NRI) was used in these analyses. CI: confidence interval. IDI: integrated discrimination improvement.

### Classification of patients with ambiguous phenotype

To test the utility of the 4 miRNA panel to improve the classification of patients with ambiguous phenotype, we considered patients with 1<WMIS<1.4 (n = 49). At 6-months follow-up, 25 patients had moderate impairment of LV contractility (1.2<WMIS<1.4) and 24 had preserved LV contractility (1<WMIS≤1.2). Logistic regression and leave-one-out cross validation were used in these analyses. Two models were built, one with clinical variables and Nt-proBNP and one with clinical variables, Nt-proBNP and the 4 miRNA panel. The model with clinical variables and Nt-proBNP had a specificity of 75%, but poor sensitivity of 48%. The 4 miRNAs panel increased the sensitivity to 60%, while maintaining the specificity at 75%. With miRNAs, the positive predictive value was increased from 67% to 71%, and the negative predictive value was increased from 58% to 64%. Therefore, the 4 miRNAs panel improved the prognostication of patients with ambiguous phenotype.

### Prediction of LV contractility

**Figure 3:**
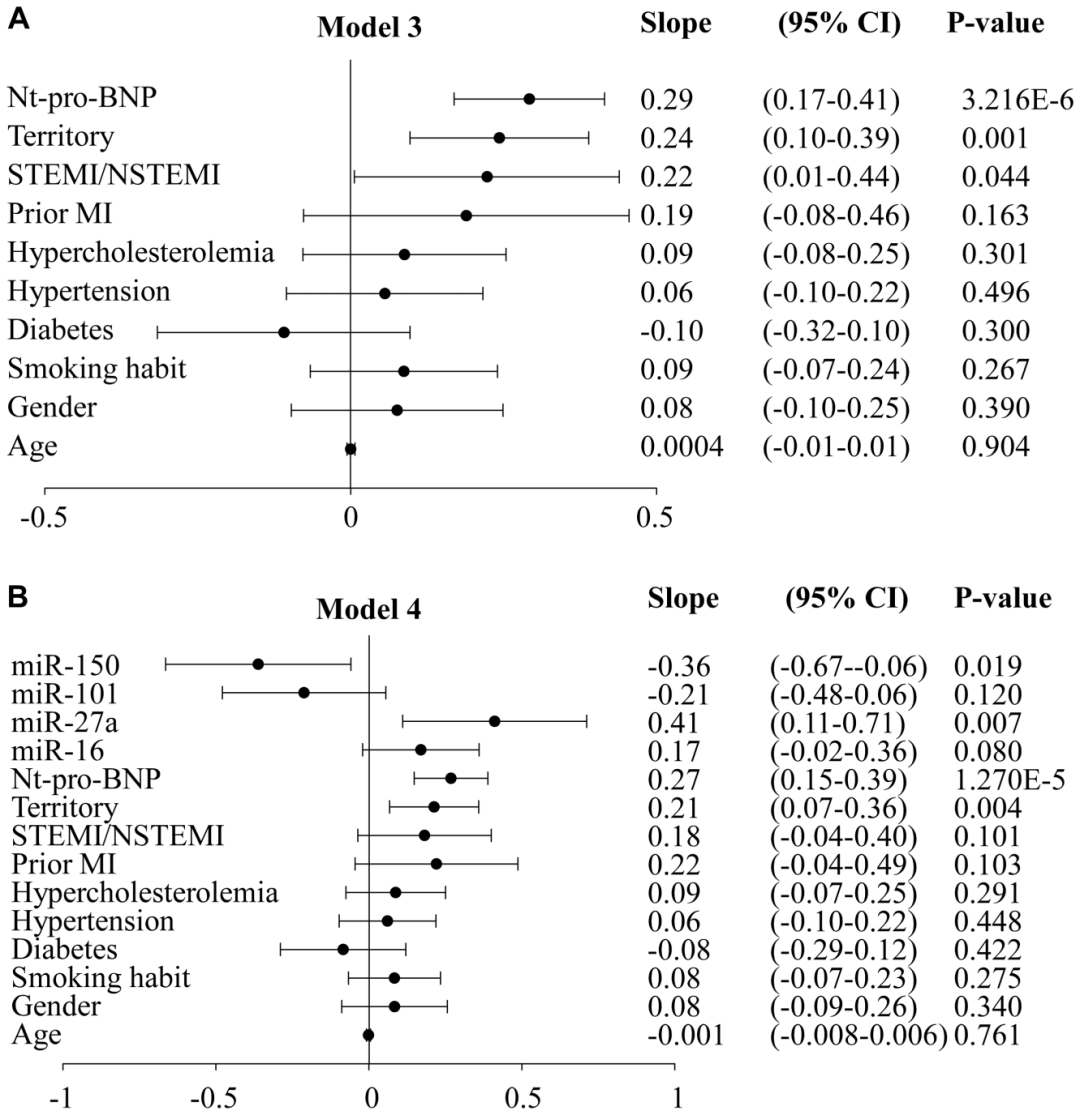
Rates of change in WMIS obtained by censored regression. Nt-proBNP and miRNAs were measured at discharge from the hospital and LV contractility was evaluated by WMIS at 6-months follow-up. Censored regression models were built to determine LV contractility. A. Model 3 is a multivariable model including indicated clinical parameters and Nt-proBNP. B. Model 4 is a multivariable model including the variables of model 3 and the expression values of miR-16/27a/101/150. CI: confidence interval.

So far, WMIS was considered as a dichotomized variable and was predicted using logistic regression models. We then investigated whether miRNAs were predictors of WMIS considered as a continuous variable. Fifty five patients had WMIS = 1, indicating fully preserved LV contractility. Due to this left censoring of WMIS values at 1, censored regression (aka “Tobit regression”) was used for prediction analysis. As for logistic regression, two models were built: model 3 includes all clinical variables and Nt-proBNP, and model 4 includes all variables of model 3 and the 4 miRNAs panel. Figure 3A shows the rates of change in WMIS by each variable in model 3. Infarct type, infarct territory and Nt-proBNP were significant predictors of WMIS. Patients with anterior STEMI and elevated Nt-proBNP had increased impaired LV contractility. Figure 3B shows that miR-27a and miR-150 were significantly related to an increased WMIS when added to model 3. The slopes of miR-16 and miR-101 were of borderline significance.

We determined the ability of each miRNA and of combinations of several miRNAs to improve the predictive value of model 3 (Table 5). While addition of all 4 miRNAs added to the predictive value of model 3 (p = 0.047), no single miRNA did so.

**Table 5:** Added value of combinations of miRNAs (censored regression).

miRNA added to model 3	Wald chi square test P-value	AIC	LRT P-value
None	4.35E-07	205.386	
miR-16	8.47E-07	206.853	0.465
miR-27a	2.66E-07	204.351	0.081
miR-101	7.38E-07	206.655	0.392
miR-150	9.69E-07	207.332	0.817
miR-16+miR-27a	6.02E-07	206.35	0.219
miR-16+miR-101	1.61E-06	208.536	0.654
miR-16+miR-150	1.10E-06	207.643	0.418
miR-27a+miR-101	3.82E-07	205.303	0.130
miR-27a+miR-150	1.68E-07	203.816	0.062
miR-101+miR-150	1.20E-06	208.074	0.519
miR-16+miR-27a+miR-101	8.31E-07	207.232	0.245
miR-16+miR-27a+miR-150	1.98E-07	204.178	0.066
miR-16+miR-101+miR-150	1.72E-06	208.95	0.487
miR-27a+miR-101+miR-150	2.49E-07	204.826	0.087
miR-16+miR-27a+miR-101+miR-150	1.51E-07	203.752	0.047

Shown are the results of all combinations of miRNAs added to model 3. The Wald chi square test indicates the overall significance of the model. The likelihood ratio test (LRT) compares the fit of a model with miRNAs to model 3. AIC: Akaike information criteria.

**Figure 4:**
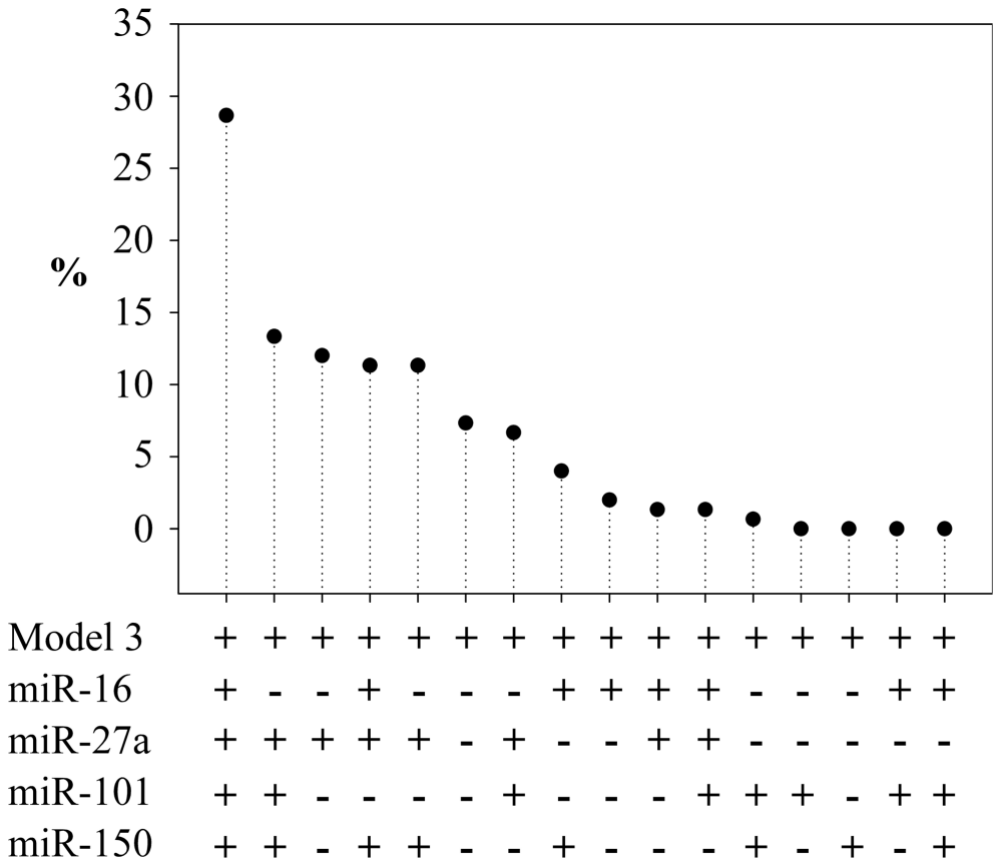
Bootstrap internal validation (censored regression). Represented is the percentage of times a combination of miRNAs was selected as providing the best improvement of the prediction of model 3 over 150 bootstrap iterations.

Bootstrap internal validation confirmed that the model including the 4 miRNAs was the best combination in 29% of the 150 iterations performed (Figure 4). MiR-27a was selected solely as the best model in 12% of cases and was included in all top models, demonstrating its contribution to the prediction.

## Discussion

In the present study, we evaluated the value of circulating miRNAs for the prediction of LV dysfunction after AMI. A panel of 4 miRNAs improved the prognostic value of a multi-variable clinical model including Nt-proBNP.

### Improvement of prognostic value of traditional markers

When added to a model containing clinical variables and Nt-proBNP, the combination of 4 miRNAs, measured prior to discharge from the index admission, improved the identification of patients who subsequently manifested impaired LV contractility. All analyses confirmed that the optimal prognostic value was obtained by the panel of 4 miRNAs. Of these, miR-27a had the strongest individual association with the development of LV systolic dysfunction at 6-month follow-up, elevated levels being associated with 15.9-fold higher risk of dysfunction. However, in censored regression analyses, only the panel of 4 miRNAs added significant prognostic value.

While previous studies investigated the prognostic value of single miRNAs after MI [7], [9], this is the first report of a panel of miRNAs which may aid in prognostication in this setting. Panels of biomarkers, from the miRNA family or others, generate an enhanced predictive value compared to single markers. In this context, Zampetaki and colleagues recently reported an incremental association between baseline expression levels of a panel of 3 miRNAs and the risk of developing MI in the 10 following years in a population-based survey [16].

In addition to improving identification of patients destined to have impaired LV function after AMI, the 4 miRNA panel improved the classification of patients with intermediate phenotype at discharge. After exclusion of patients who at hospital discharge had fully preserved LV contractility (WMIS = 1) or severe contractile dysfunction (WMIS>1.4), the 4 miRNA panel improved the prediction of impaired LV contractility at 6-month follow-up, when added to Nt-proBNP and clinical parameters. The sensitivity of the prediction was improved, and the specificity was preserved. This observation is clinically relevant in view of the difficulty to classify patients with intermediate phenotype.

### Functional roles of miRNAs in LV remodelling

It is now clear that miRNAs play active roles in multiple pathways leading to LV remodelling [17]. Three of the 4 miRNAs studied in the present study, miR-27a/101/150, were chosen from a systems-biology approach aimed at identifying miRNAs likely to regulate the expression of genes associated with LV remodelling [10]. The choice of these 3 miRNAs relies on their demonstrated association with LV remodelling [10]. miR-16 was also selected following our finding of its association with key players of LV remodelling [18]. We previously verified that miR-150 regulates LV remodelling, through inhibition of the expression of C-reactive protein and adrenergic receptor beta 1 [10]. Multiple miRNAs are dys-regulated after AMI and during cardiac hypertrophy [19], [20]. Among these, miR-27a is up-regulated and decreases expression of peroxisome proliferator-activated receptor gamma, an inhibitor of cardiomyocytes hypertrophy [21], thereby resulting in activation of hypertrophic signals. While cardiac-enriched miR-1 and miR-133 are known to promote arrhythmogenesis [22], [23], few studies demonstrated the involvement of miRNAs in modulation of cardiac contractility [24]. A report showing that miR-27a up-regulates beta-myosin heavy chain expression in cardiac myocytes, through repression of the thyroid receptor beta 1 gene, suggested that miR-27a may stimulate LV contractility [25]. A recent report demonstrated that miR-101 inhibits fibrosis and thus may help preserve LV function after MI [26], conferring a cardioprotective role for this miRNA.

In the current report, Nt-proBNP, miR-16, and miR-27a were positively associated with development of LV dysfunction (odds-ratio>1), in contrast to miR-150 and miR-101 (odds-ratio<1). These observations are consistent with our previous observation that circulating levels of miR-150 are higher in AMI patients without LV dilatation [10], and with data from other groups showing that miR-27a is up-regulated during cardiac hypertrophy [21]. On the other hand, other groups suggested that miR-27a may stimulate contractility [25], [27]. Further studies are required to investigate the functional roles of miRNAs in the development of LV remodelling, as well as their potential as therapeutic target.

### Methodological discussion

Our first analyses were performed by logistic regression after dichotomization of patients according to WMIS value. Although a common approach, dichotomization induces loss of information, which can be significant [28]. According to Fedorov et al., for normally distributed data, this loss of information is at least 36% as compared to a situation where the outcome variable is used in its continuous form [29].

Since WMIS is censored at 1 (i.e. 37% of patients were attributed a WMIS of 1, i.e. fully preserved LV contractility), we used censored regression instead of linear regression [13]. In this study, no nonlinear relationships or large deviations from normality were observed when plotting the models residuals. In a more general framework, restricted cubic splines or fractional polynomials could be used to account for these issues. Both censored and logistic regression confirmed the additive value of the 4 miRNAs panel to a multi-parameter clinical model including the gold-standard Nt-proBNP.

Finally, to avoid potential over-fitting due to inclusion of a large number of predictive variables, the AIC was used in statistical analyses as a criterion to select models, and the results were validated by bootstrap.

### Limitations

Our study is observational and uses WMIS as a surrogate for prognosis. However, residual LV function is an important determinant of prognosis after AMI [30], and we have demonstrated the association of WMIS with adverse outcome in this cohort [11]. Moreover, our cohort of patients with impaired LV contractility, identified with our miRNA panel, did show elevated risk of developing heart failure after discharge. Coronary reperfusion therapy was utilised in a relatively small proportion of our historical cohort. However, even when included in multivariate model for prediction of WMIS, reperfusion therapy did not alter in any meaningful way the predictive value of miRNA expression. Since reperfusion rate was relatively low in this cohort, and was not included in predictive variables, the prognostic utility of the miRNA panel should be confirmed in a larger cohort of patients managed in the contemporary era of primary percutaneous coronary intervention. Of note, the prognostic value of miRNAs was evident only in combination, not when considered individually.

### Conclusion

A panel of miRNAs has the potential to improve the identification of patients at risk of adverse LV remodelling following STEMI. Further studies are required to confirm the prognostic value of miRNAs in this setting, and in the setting of other cardiac conditions.

## References

[1] Torabi A, Cleland JG, Khan NK, Loh PH, Clark AL, et al (2008) The timing of development and subsequent clinical course of heart failure after a myocardial infarction. Eur Heart J 29: 859–870. 18353754 10.1093/eurheartj/ehn096

[2] Talwar S, Squire IB, Downie PF, McCullough AM, Campton MC, et al (2000) Profile of plasma N-terminal proBNP following acute myocardial infarction; correlation with left ventricular systolic dysfunction. Eur Heart J 21: 1514–1521. 10973765 10.1053/euhj.1999.2045

[3] Gilad S, Meiri E, Yogev Y, Benjamin S, Lebanony D, et al (2008) Serum microRNAs are promising novel biomarkers. PLoS One 3: e3148. 18773077 10.1371/journal.pone.0003148 PMC2519789

[4] Mitchell PS, Parkin RK, Kroh EM, Fritz BR, Wyman SK, et al (2008) Circulating microRNAs as stable blood-based markers for cancer detection. Proc Natl Acad Sci U S A 105: 10513–10518. 18663219 10.1073/pnas.0804549105 PMC2492472

[5] Creemers EE, Tijsen AJ, Pinto YM (2012) Circulating MicroRNAs: Novel Biomarkers and Extracellular Communicators in Cardiovascular Disease? Circ Res 110: 483–495. 22302755 10.1161/CIRCRESAHA.111.247452

[6] Corsten MF, Dennert R, Jochems S, Kuznetsova T, Devaux Y, et al (2010) Circulating MicroRNA-208b and MicroRNA-499 reflect myocardial damage in cardiovascular disease. Circ Cardiovasc Genet 3: 499–506. 20921333 10.1161/CIRCGENETICS.110.957415

[7] Devaux Y, Vausort M, Goretti E, Nazarov PV, Azuaje F, et al (2012) Use of Circulating MicroRNAs to Diagnose Acute Myocardial Infarction. Clin Chem 58: 559–567. 22252325 10.1373/clinchem.2011.173823

[8] Zile MR, Mehurg SM, Arroyo JE, Stroud RE, Desantis SM, et al (2011) Relationship Between The Temporal Profile of Plasma microRNA and Left Ventricular Remodeling In Patients Following Myocardial Infarction. Circ Cardiovasc Genet 4: 614–619. 21956146 10.1161/CIRCGENETICS.111.959841 PMC3535326

[9] Widera C, Gupta SK, Lorenzen JM, Bang C, Bauersachs J, et al (2011) Diagnostic and prognostic impact of six circulating microRNAs in acute coronary syndrome. J Mol Cell Cardiol 51: 872–875. 21806992 10.1016/j.yjmcc.2011.07.011

[10] Devaux Y, Vausort M, McCann G, Zangrando J, Kelly D, et al (2013) MicroRNA-150: A Novel Marker of Left Ventricular Remodeling After Acute Myocardial Infarction. Circ Cardiovasc Genet April 1. DOI /circgenetics 10.1161/CIRCGENETICS.113.000077 23547171

[11] Kelly D, Cockerill G, Ng LL, Thompson M, Khan S, et al (2007) Plasma matrix metalloproteinase-9 and left ventricular remodelling after acute myocardial infarction in man: a prospective cohort study. Eur Heart J 28: 711–718. 17339265 10.1093/eurheartj/ehm003 PMC2202923

[12] Omland T, Persson A, Ng L, O'Brien R, Karlsson T, et al (2002) N-terminal pro-B-type natriuretic peptide and long-term mortality in acute coronary syndromes. Circulation 106: 2913–2918. 12460871 10.1161/01.cir.0000041661.63285.ae

[13] Tobin J (1958) Estimation of relationships for limited dependent variables. Econometrica 26: 24–36.

[14] Pencina MJ, D'Agostino RB Sr, D'Agostino RB Jr, Vasan RS (2008) Evaluating the added predictive ability of a new marker: from area under the ROC curve to reclassification and beyond. Stat Med 27: 157–172 discussion 207–112. 17569110 10.1002/sim.2929

[15] Steyerberg EW, Harrell FE Jr, Borsboom GJJM, Eijkemans MJC, Vergouwe Y, et al (2001) Internal validation of predictive models: Efficiency of some procedures for logistic regression analysis. J Clin Epidemiol 54: 774–781. 11470385 10.1016/s0895-4356(01)00341-9

[16] Zampetaki A, Willeit P, Tilling L, Drozdov I, Prokopi M, et al (2012) Prospective Study on Circulating MicroRNAs and Risk of Myocardial Infarction. J Am Coll Cardiol 60: 290–299. 22813605 10.1016/j.jacc.2012.03.056

[17] Zhu H, Fan GC (2012) Role of microRNAs in the reperfused myocardium towards post-infarct remodelling. Cardiovasc Res 94: 284–292. 22038740 10.1093/cvr/cvr291 PMC3331611

[18] Goretti E, Rolland-Turner M, Léonard F, Zhang L, Wagner DR, et al (2013) MicroRNA-16 affects key functions of human endothelial progenitor cells. J Leukoc Biol In press. 10.1189/jlb.1012511 23325924

[19] Abdellatif M (2012) Differential Expression of MicroRNAs in Different Disease States. Circ Res 110: 638–650. 22343558 10.1161/CIRCRESAHA.111.247437 PMC3324925

[20] Da Costa Martins PA, De Windt LJ (2012) MicroRNAs in control of cardiac hypertrophy. Cardiovasc Res 93: 563–572. 22266752 10.1093/cvr/cvs013

[21] Yamamoto K, Ohki R, Lee RT, Ikeda U, Shimada K (2001) Peroxisome Proliferator-Activated Receptor γ Activators Inhibit Cardiac Hypertrophy in Cardiac Myocytes. Circulation 104: 1670–1675. 11581147 10.1161/hc4001.097186

[22] Belevych AE, Sansom SE, Terentyeva R, Ho HT, Nishijima Y, et al (2011) MicroRNA-1 and -133 Increase Arrhythmogenesis in Heart Failure by Dissociating Phosphatase Activity from RyR2 Complex. PLoS ONE 6: e28324. 22163007 10.1371/journal.pone.0028324 PMC3232211

[23] Yang B, Lin H, Xiao J, Lu Y, Luo X, et al (2007) The muscle-specific microRNA miR-1 regulates cardiac arrhythmogenic potential by targeting GJA1 and KCNJ2. Nat Med 13: 486–491. 17401374 10.1038/nm1569

[24] Aurora AB, Mahmoud AI, Luo X, Johnson BA, van Rooij E, et al (2012) MicroRNA-214 protects the mouse heart from ischemic injury by controlling Ca2+ overload and cell death. The Journal of Clinical Investigation 122: 1222–1232. 22426211 10.1172/JCI59327 PMC3314458

[25] Nishi H, Ono K, Horie T, Nagao K, Kinoshita M, et al (2011) MicroRNA-27a Regulates Beta Cardiac Myosin Heavy Chain Gene Expression by Targeting Thyroid Hormone Receptor β1 in Neonatal Rat Ventricular Myocytes. Molecular and Cellular Biology 31: 744–755. 21149577 10.1128/MCB.00581-10 PMC3028640

[26] Pan Z, Sun X, Shan H, Wang N, Wang J, et al (2012) miR-101 Inhibited Post-Infarct Cardiac Fibrosis and Improved Left Ventricular Compliance via FOS/TGFβ1 Pathway. Circulation July 18. 10.1161/CIRCULATIONAHA.112.094524 22811578

[27] Sadegh MK, Ekman M, Rippe C, Uvelius B, Swärd K, et al (2012) Deletion of Dicer in Smooth Muscle Affects Voiding Pattern and Reduces Detrusor Contractility and Neuroeffector Transmission. PLoS ONE 7: e35882. 22558254 10.1371/journal.pone.0035882 PMC3338793

[28] Senn S, Julious S (2009) Measurement in clinical trials: a neglected issue for statisticians? Stat Med 28: 3189–3209. 19455540 10.1002/sim.3603

[29] Fedorov V, Mannino F, Zhang R (2009) Consequences of dichotomization. Pharm Stat 8: 50–61. 18389492 10.1002/pst.331

[30] Mann DL, Barger PM, Burkhoff D (2012) Myocardial Recovery and the Failing Heart: Myth, Magic, or Molecular Target? J Am Coll Cardiol 60: 2465–2472. 23158527 10.1016/j.jacc.2012.06.062 PMC3522780

