# Correction: A Panel of 4 microRNAs Facilitates the Prediction of Left Ventricular Contractility after Acute Myocardial Infarction

**DOI:** 10.1371/annotation/458a1f6a-6327-429a-81cb-992c97f04bd6

**Published:** 2013-08-23

**Authors:** Yvan Devaux, Melanie Vausort, Gerry P. McCann, Dominic Kelly, Olivier Collignon, Leong L. Ng, Daniel R. Wagner, Iain B. Squire

There was an error in the Funding statement. The correct version of the statement is available below:

This work was supported by the Society for Research on Cardiovascular Diseases, the Ministry of Culture, Health and Higher Education, and the National Fund for Research of Luxembourg. Profs. Ng and Squire and Dr. McCann received support from the NIHR Leicester Cardiovascular Biomedical Research Unit. The funders had no role in study design, data collection and analysis, decision to publish, or preparation of the manuscript. 

